# Tunable Mechanical Metamaterials through Hybrid Kirigami Structures

**DOI:** 10.1038/s41598-018-21479-7

**Published:** 2018-02-21

**Authors:** Doh-Gyu Hwang, Michael D. Bartlett

**Affiliations:** Department of Materials Science and Engineering, Soft Materials and Structures Lab, Iowa State University of Science and Technology, 528 Bissell Rd, Ames, IA 50011 USA

## Abstract

Inspired by the art of paper cutting, kirigami provides intriguing tools to create materials with unconventional mechanical and morphological responses. This behavior is appealing in multiple applications such as stretchable electronics and soft robotics and presents a tractable platform to study structure-property relationships in material systems. However, mechanical response is typically controlled through a single or fractal cut type patterned across an entire kirigami sheet, limiting deformation modes and tunability. Here we show how hybrid patterns of major and minor cuts creates new opportunities to introduce boundary conditions and non-prismatic beams to enable highly tunable mechanical responses. This hybrid approach reduces stiffness by a factor of ~30 while increasing ultimate strain by a factor of 2 (up to 750% strain) relative to single incision patterns. We present analytical models and generate general design criteria that is in excellent agreement with experimental data from nanoscopic to macroscopic systems. These hybrid kirigami materials create new opportunities for multifunctional materials and structures, which we demonstrate with stretchable kirigami conductors with nearly constant electrical resistance up to >400% strain and magnetoactive actuators with extremely rapid response (>10,000% strain s^−1^) and high, repeatable elongation (>300% strain).

## Introduction

The ability to tune the stiffness and deformation behavior of materials is critical to diverse applications from stretchable electronics and soft robotics to tissue engineering and biomedical devices^1–7^. Recently, techniques building upon principles in kirigami, the Japanese art of paper cutting, have demonstrated that the addition of cuts to materials enables elastic softening, large deformations, and the generation of 3D structures from 2D sheets across a range of length scales^8–15^. This enables the utilization of inextensible or functional components to create deformable devices such as reconfigurable electronics, optoelectronics, and sensors^16–27^. As kirigami features generate geometric deformations that dominate the inherent material elasticity, properties and structures of kirigami metamaterials are controlled by the pattern and orientation of the cuts. This has been examined by recent efforts to concentrate deformations on either the bending of beams defined by cuts or by the rotation of hinges that separate these features^28–35^. However, exploring the synergistic coupling of these deformation modes has been limited and further cut architecture modification has the potential to provide significant gains in stiffness tunability and deformability in kirigami metamaterials.

Here we show that the addition of minor cuts to kirigami structures provides a mechanism to control kirigami deformation modes for highly tunable mechanical response. We investigate this approach in polymeric films by adding minor cuts near the ends of major cuts to prescribe boundary conditions and create non-prismatic kirigami beams. This reduces stiffness by a factor of 30 and increases ultimate tensile strain by a factor of 2 relative to single incision patterns. Experimental results are supported by theoretical predictions in which the addition of minor cuts of various lengths and proximity to major cuts provides an analogous response to varying boundary conditions and beam geometry. We generalize these equations and provide design rules for kirigami films with tunable stiffness and extension responses and find excellent agreement between predictions and experimental measurements from nanoscopic to macroscopic kirigami materials. This approach and corresponding design criteria provide a simple and scalable method for the control of stiffness and deformation of materials without changing sample size, major pattern geometry, or material composition. We demonstrate the utility of this approach in stretchable electronics with highly stretchable conductors for deformable kirigami circuitry and in programmable matter with a rapid magnetoactive soft actuator. Both examples show that minor cuts enable a significant enhancement in deformability and stiffness control.

## Results

**Figure 1:**
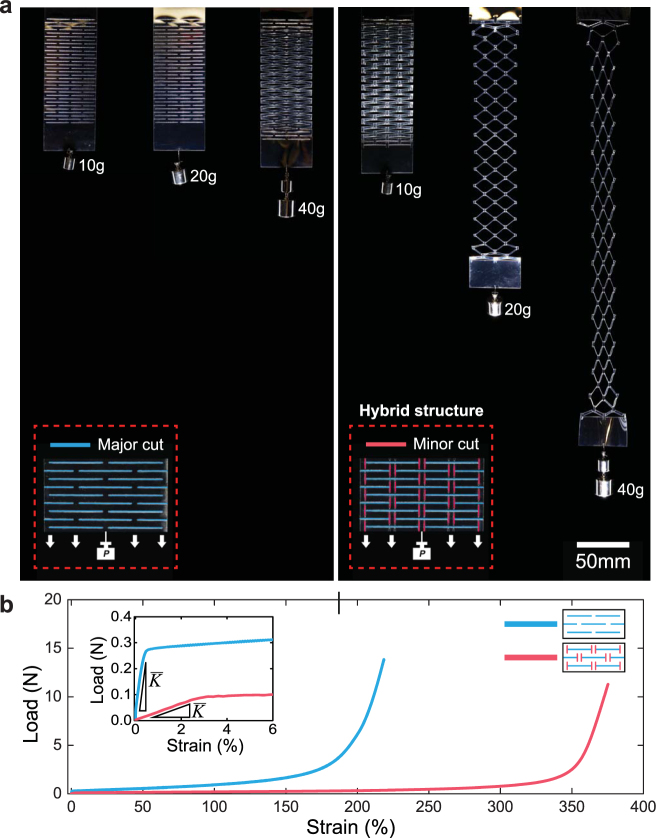
Tunable kirigami materials through hybrid structures. (**a**) Photographs of kirigami sheets with (left) only major cuts (Fig. 2a (design *i*)) and (right) hybrid structures of major and minor cuts (Fig. 2a (design *v*)). The same increasing weights (10 g, 20 g, and 40 g) are attached to each kirigami sheet to demonstrate mechanical response. Major cuts are highlighted in blue and the minor cuts are highlighted in red in the insets. (**b**) Load (*P*) versus strain (*ε*) for kirigami materials with design *i* of *w* = 3 mm (blue curve) and with design *v* of *w* = 3 mm and *l*_*m*_/2*w* = 0.75 (red curve). Geometric parameters are described in Fig. 2a. The inset shows the initial regime of the *P* - *ε* plot and the slope indicates the effective in-plane stiffness.

Kirigami patterns are created in polyethylene terephthalate (PET) sheets (*E* ≈ 2.6 GPa) with a CO_2_ laser cutter. The patterned cuts consist of an array of transverse long major cuts, which define beams, and longitudinal short minor cuts, which prescribe beam shape and the rigidity of boundary conditions between beams. As seen in Fig. 1a, the addition of minor cuts significantly modifies the deformation behavior of kirigami film, resulting in a lower stiffness and higher elongation than a kirigami film consisting only of major cuts. Specifically, when hanging weights, a kirigami system consisting of only major cuts shows negligible strain (*ε* ≈ 0%) until the force reaches 0.4 N. In contrast, a modified system with major and minor cuts begins extending at a far lower force (≈0.1 N) and reaches strains over 270% at a force of 0.4 N. The minor cuts also increase the ultimate extensibility of the films, as seen in Fig. 1b.

**Figure 2:**
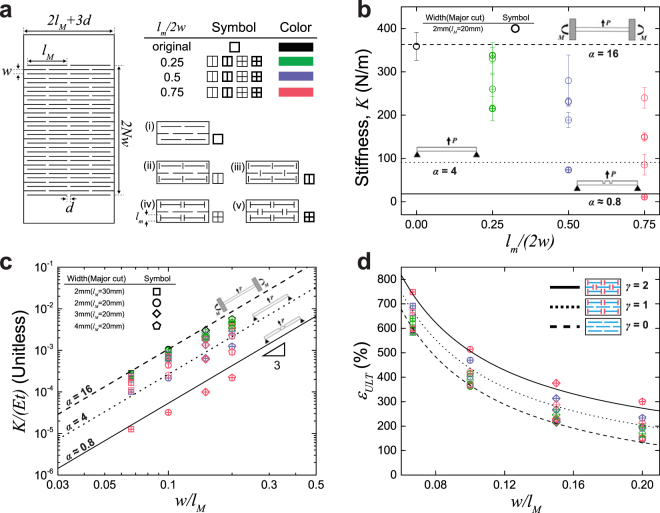
Hybrid kirigami stiffness and ultimate strain. (**a**) A schematic of a kirigami structure with geometric parameters and different designs. Data point symbol represents each beam size, which is defined by major cuts, data point interior represents different minor cut designs, and data point color represents the ratio of minor cut length over width of beam. (**b**) Effective in-plane stiffness $\overline{K}$ versus *l*_*m*_/(2*w*) for varying pattern designs, with *l*_*M*_ = 20 mm, *w* = *d* = 2 mm, and *l*_*m*_ = 0–3 mm. Dashed/dotted/solid lines represent Equation 2 with FFE (*α* = 16), PPE (*α* = 4), and non-prismatic beam/pinned end condition (*α* ≈ 0.8), respectively. (**c**) $\overline{K}/(Et)$ versus (*w*/*l*_*M*_) with varying major and minor cut conditions, dashed/dotted/solid lines represent the same *α* values as in component (**c**). (**d**) Ultimate strain (*ε*_*ULT*_) versus *w*/*l*_*M*_, where lines represent Equation 3 with various *γ* values as specified in the legend.

We consider the kirigami sheet as an array of slender beams with rectangular cross-section defined by the major cuts. Each beam is characterized by length *l*_*M*_, width *w*, and thickness *t* with elastic modulus *E*. The spacing *d* between major cuts along the transverse direction and the thickness of samples are constant throughout the experiment (*d* = 2 mm, *t* = 0.13 mm). The length of minor cuts (*l*_*m*_) is selected according to the dimensionless geometric parameter *l*_*m*_/2*w* = 0, 0.25, 0.5, 0.75 and five different minor cut arrangements are investigated: (i) no minor cuts, (ii) minor cuts in between major cuts in alternating rows and (iii) all rows, (iv) minor cuts intersecting major cuts in alternating rows and v) all rows (Fig. 2a). Figure 1b shows a representative force-extension plot of a patterned sheet with and without minor cuts. Initially, the patterned sheet deforms with each beam bending in plane through the opening of cuts and rotation of plain regimes via hinges. As extension increases the in-plane beam bending becomes energetically more costly than out-of-plane bending^28,30^, resulting in an elastic instability and a transition to out-of-plane beam bending (See Fig. S1 for close-up images of the structure under low strain). This continues until the deformation fully extends each beam, at which point the structure becomes loaded in tension, resulting in strain hardening and ultimately failure.

To analyze the mechanical behavior of these kirigami structures we consider an array of slender beams, where the stiffness of a single beam scales as $k\cong E{w}^{3}t/{l}_{M}^{3}$, where *E* is the elastic modulus and *l*_*M*_, *w*, and *t* are length, width, and thickness of the beam, respectively^28^. The effective in-plane stiffness $\overline{K}$ of the patterned sheet is calculated by considering of the number of beams and their arrangement (*N*_*B*_/*N*_*rows*_) such that:

1 $$\overline{K}=\alpha \frac{{N}_{B}}{{N}_{rows}}\frac{E{w}^{3}t}{{l}_{M}^{3}}$$

where *N*_*B*_ is the number of beams in each row, *N*_*rows*_ is the number of rows along the loading direction, and *α* is a numerical coefficient which is dependent on boundary conditions and beam shape. As the major cuts define the primary beam dimensions *l*, *w*, *t*, minor cuts are introduced to explore the tunability of *α*. Figure 2b illustrates the stiffness dependence of a kirigami sheet on minor cuts as a function of the dimensionless minor cut parameter *l*_*m*_/(2*w*). Here, a kirigami sheet without minor cuts shows the highest stiffness while as minor cuts are introduced the stiffness decreases as *l*_*m*_/(2*w*) increases. Additionally, at each value of *l*_*m*_/(2*w*), stiffness decreases as the minor cuts intersect the major cuts and as the number of minor cuts increases. We consider that in the absence of minor cuts the ends of each beam are restrained from rotation and the in-plane bending occurs through the opening of major cuts and is analogous to a fixed-fixed end condition (FFE). As minor cuts are introduced near beam ends the FFE boundary conditions relax as the hinge regions are softened and can no longer support bending moments resulting in a transition to pinned-pinned end conditions (PPE). This leads to deformations through the opening of major and minor cuts. To quantitatively describe this behavior the numerical coefficient *α* is calculated and is found to range between FFE (*α* = 16) and PPE condition (*α* = 4) (See Fig. S2 for derivations). When these bounds are plotted in Fig. 2b it is seen that the data falls within the bounds of these conditions for design *i*-*iv*, supporting the analogy of minor cuts to beam boundary conditions.

We further explore beam geometry by applying a kirigami design to create non-prismatic beams (Figs 2a(v) and S3). In most cases, non-prismatic beams with varying moments of inertia along the length are employed for enhancing mechanical strength and stiffness^36^. In contrast, we utilize this approach in the kirigami system to further reduce stiffness and obtain an *ultra*-*soft kirigami* structure. This is achieved by introducing minor cuts onto the beam midspan without changing major cut length. The design decreases the area moment of inertia in regions where the bending moment is largest, resulting in an enhanced softening effect on the PPE condition. Upon calculating, the numerical coefficient *α* is ≈0.8 (Fig. S3), which is lower than the PPE condition (*α* = 4), and is consistent with the trend in the data in Fig. 2b.

Upon rearrangement of equation (1) the relation between effective stiffness and geometric parameters is $\overline{K}/Et=\alpha \tfrac{{N}_{B}}{{N}_{rows}}{(w/{l}_{M})}^{3}$. Figure 2c presents a log-log plot of our experimental data with 52 combinations of major (two different lengths *l*_*M*_ = 20 mm, 30 mm and three different widths *w* = 2 mm, 3 mm, and 4 mm) and minor cuts and shows that the data for prismatic beams collapses into the range bounded by FFE and PPE conditions. Some deviation from this prediction is observed as *l*_*M*_ ≤ 5*w*, which was also observed by Isobe *et al*.^28^, as the beam begins to violate the slender beam assumption. The stiffness is greatly reduced by constructing kirigami structures with non-prismatic beams. When design (v) with 0.75 *l*_*m*_/(2*w*) is adopted, which shows the smallest moment of inertia in the midspan of our designs, the stiffness decreases by a factor of ≈22–30 compared to systems consisting only of major cuts. The stiffness data for this non-prismatic beam continues to follow the scaling prediction with *α* ≈ 0.8. Additionally, the *ultra*-*soft kirigami* structure shows significantly reduced stiffness compared to a design with just major cuts throughout the entire strain range (see Fig. S4). These dramatic changes in stiffness through the *α* parameter by controlling boundary conditions and moment of inertia along the beam greatly increases design flexibility of kirigami materials, especially in systems with size limitations or fabrication constraints.

In addition to the control of initial stiffness, kirigami structures can also tune the ultimate strain (*ε*_*ULT*_) of materials. Figure 2d illustrates the ultimate strain of each system with different geometries and arrays of minor cuts as a function of a dimensionless major cut parameter *w*/*l*_*M*_. As the width-length ratio *w*/*l*_*M*_ decreases, the ultimate strain increases as beams become relatively longer with regards to their cross-section (*wt*), resulting in larger axial displacements. In addition, the ultimate strain is affected by the array of minor cuts. The lowest ultimate strain is achieved utilizing only major cuts, whereas the highest is achieved for hybrid cuts with design *v* and *l*_*m*_/(2*w*) = 0.75. To predict ultimate strain where kirigami sheets break, we derive a model based on beam geometry at ultimate strain. Here, we assume *w*, $d\ll {l}_{M}$ and expand previous models^29^ to include minor cuts and the reduction of *ε*_*ULT*_ by edge effects such that:

2 $${\varepsilon }_{ULT}\cong \frac{({N}_{rows}-\mathrm{2)}\,({l}_{M}+\gamma {l}_{m})}{2{N}_{rows}w}-1$$

where *γ* is a numerical coefficient to account for the contribution of minor cuts to *ε*_*ULT*_; *γ* = 0 in the absence of minor cuts (design *i*), *γ* = 1 or 2 in the presence of minor cuts in alternating rows (design *iv*) or every row (design *v*), respectively (Full derivation in Figs S5 and S6). Upon plotting *ε*_*ULT*_ as a function of *w*/*l*_*M*_, we find excellent agreement between the experimental data and Equation 2. When minor cuts are included it enables further rotation of hinge regions by opening of minor cuts, as indicated by the increase in *ε*_*ULT*_ for a fixed value of beam aspect ratio (*w*/*l*_*M*_). Further, we find that the force at break of low to moderate aspect ratio beams (*w*/*l*_*M*_ ≥ 0.15) is not significantly influenced by minor cuts, with a slight decrease for very slender beams (*w*/*l*_*M*_ ≤ 0.1) (Fig. S7), which we attribute to the thin hinge regions. To increase the force capacity of these films the ends of the cuts could be modified with rounded or engineered corners, which has previously been demonstrated to reduce stress concentrations and delay fracture^34^. Thus, minor cuts provide an additional means to increase and tune the strain at break for kirigami materials.

## Discussion

To provide a general relationship for the design of kirigami structures consisting of arrays of beams for high extensibility and low stiffness, we combine Equations 1 and 2 such that:

3 $$\frac{{\varepsilon }_{ULT}}{\overline{K}}=\frac{1}{E\alpha }\,(\frac{{N}_{rows}}{{N}_{B}})\,(\frac{{l}_{M}+\gamma {l}_{m}}{2w}-1)\,\frac{{l}_{M}^{3}}{{w}^{3}t}$$

Equation 3 shows that the mechanical response of kirigami structures is controlled by the geometry (*l*_*M*_, *w*, *t*) and arrangement (*N*_*rows*_, *N*_*B*_) of beams, which are defined by major cuts, the arrangement/length of minor cuts (*α*, *l*_*m*_, *γ*), and the elastic properties (*E*) of the film. The *ε*_*ULT*_/$\overline{K}$ metric presented in Equation 3 is useful for understanding the soft mechanical response of materials. Here, materials with high extensibility and low stiffness will give large values of ${\varepsilon }_{ULT}/\overline{K}$, providing a means to compare material compliance. This is useful for fields such as stretchable and wearable electronics and soft robotics, where relative compliance can be used as a material selection tool^37^.

**Figure 3:**
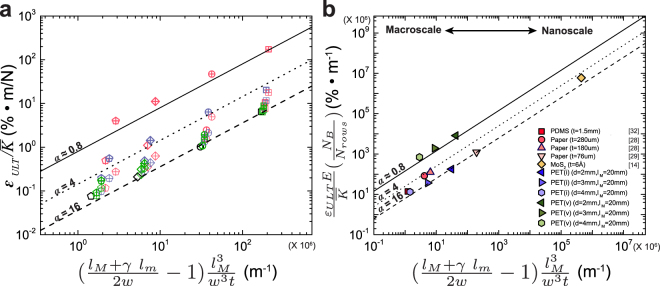
Generalized design criteria across length scales. (**a**) Mechanical property ${\varepsilon }_{ULT}/\overline{K}$ versus a combined geometric parameter for all the data in this work, where lines represent Equation 3. (**b**) Scaling plot showing agreement between Equation 3 and experimental/simulation data from kirigami materials in the literature and from the current work, across a wide range of length scales and material classes.

Upon plotting ${\varepsilon }_{ULT}/\overline{K}$ as a function of major and minor cut geometry in Fig. 3a, we find good agreement between the predictions and experimental data, where *α* provides a mechanism to tune the ratio of ${\varepsilon }_{ULT}/\overline{K}$ for a fixed beam geometry. As this equation is composed of geometric and material properties we expect the predictions to function over a wide range of scales from nanoscopic to macroscopic size scales. By rearranging Equation 3 to normalize for the number of beams in the system, we plot diverse experimental and simulation data from kirigami studies in literature along with our data in Fig. 3b. We observe that all the data with only major patterns collapse into a region bounded by *α* = 4 and *α* = 16 while the addition of minor cuts is captured with *α* = 0.8. This analysis describes the behavior of kirigami materials from sub-nanometer to millimeter film thickness with diverse material classes. Although nano-scale effects may influence the scaling at nanometer dimensions, the presented results and analysis provide evidence for the scalability of kirigami materials across tremendous length scales. Further experimentation with nano-scale 2D materials, such as graphene^8^, could be utilized as model systems to further explore the theoretical predictions at small scales.

**Figure 4:**
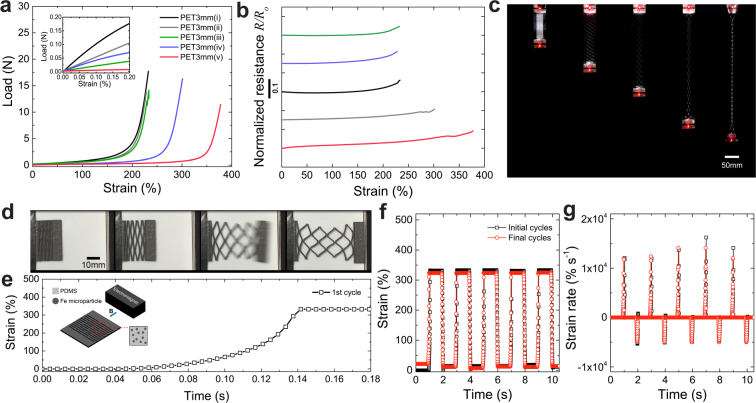
Multifunctional materials through hybrid kirigami materials. (**a**) Load versus strain plot for kirigami stretchable electrodes with various hybrid designs. The inset shows the tunable stiffness in the initial regime (*l*_*M*_ = 20 mm, *l*_*m*_ = 4.5 mm, *w* = 3 mm). (**b**) Normalized resistance versus strain plot showing that the electrical resistance for each design remains largely constant through failure. (**c**) Demonstration of hybrid kirigami structures as stretchable interconnects under axial deformation up to 380% strain. (**d**) High speed photographs of a magnetoactive hybrid kirigami structure made of Fe-PDMS with design *v* (*l*_*M*_ = 15 mm, *l*_*m*_ = 1.3 mm, *w* = 0.9 mm, and *t* = 0.82 mm) in response to a magnetic field. (**e**) Strain versus time plot of the patterned sample during the first cycle and (**f**) during the initial cycling and after ~1000 cycles (final cycles) maintaining a large deformation (~330%). (**g**) Strain rate versus time plot of the sample during the initial cycles and final cycles, showing rapid motion in response to a magnetic field.

As our kirigami films are mechanically compliant and highly deformable, they enable opportunities to create multifunctional materials for soft and stretchable electronics and components for soft robotics. To investigate their use as stretchable conductors we add a conductive liquid metal electrode on top of kirigami films with a range of minor cut conditions and measure electrical resistance as a function of strain. We find that the electrical resistance of all the samples remains largely unchanged upon deformation, but the addition of minor cuts allows the stiffness and ultimate strains to be tuned (Fig. 4). In the case of an ultra-soft kirigami film, the electrical resistance increases by less than 10% up to a strain of 380%. The viability of kirigami as stretchable interconnects is demonstrated with a LED circuit (Fig. 4c), where the addition of minor cuts allows for strains up to ~400%, compared to ~230% with only major cuts. The LED is illuminated up to the breaking strain, showing the potential to create kirigami interconnects with easily tunable stiffness and deformation properties for stretchable electronic applications.

Furthermore, we show how our kirigami materials can be used as actuators or reconfigurable matter by creating magnetoactive soft actuators. Samples are fabricated by embedding Fe microparticles into a matrix of polydimethylsiloxane (PDMS) and then laser machining kirigami patterns into the cured composite. When a kirigami actuator with hybrid cut patterns (design *v*) is activated by an electromagnet, the sheet rapidly elongates to 330% strain in ~0.1 s with a maximum strain rate during the cycle greater than 10,000% s^−1^ (Fig. 4d,e and Video S1). By alternating the magnetic field on/off at 1 s intervals, the hybrid kirigami actuator is activated and released over 1,000 cycles without degradation in speed or stroke (Fig. 4f,g). This approach to adaptive soft actuators enables an unique and exceptional combination of high-speed actuation, large deformation, and stable reversibility, outperforming nearly all other soft matter actuators and morphing technologies^38,39^. By further tuning hybrid cut structures and material choice the actuators can respond and adapt to different stimuli across a range of length scales, providing a rich design space for multifunctional materials and structures.

The synergistic mechanical coupling of hybrid major and minor cuts in kirigami materials enables highly tunable mechanical metamaterials. These hybrid structures can introduce effective boundary conditions and non-prismatic structures which can be quantitatively captured by the numerical coefficient *α*, which in the current work was varied in the range of 0.8 ≤ *α* ≤ 16. However, *α* can be further controlled and decreased to create softer kirigami structures by increasing the length and number of minor cuts to further increase the proportion of regions where the moment of inertia is small. This approach can be used to increase the mechanical response of materials to external stimuli such as mechanical force, magnetic or electrical fields, or chemical triggers. We show how these hybrid structures can be used to create ultra-soft and highly deformable multifunctional materials such as stretchable conductors with nearly invariable resistance to extreme strain and actuators with rapid response and high elongation. The mechanical response of hybrid kirigami materials is well captured by our analytical expressions and leads to compact design criteria which can be used to efficiently create kirigami-based materials for diverse applications across a wide range of length scales.

## Methods

### Materials and mechanical measurements

Kirigami sheets are prepared by laser cutting (Epilog Laser Fusion M2, 75 watt) PET films (Grainger, *E* = 2.6 ± 0.1 GPa). The elastic modulus of pristine PET films is calculated from the initial, linear regime ($\varepsilon \ll \mathrm{1 \% }$) of a force-extension plot, captured with a 10 kN load cell on an Instron 5944. The kirigami patterns on the PET film consist of slender beams (*l*_*M*_ = 20, 30 mm) with rectangular cross-section (*w* = 2, 3, 4 mm, *t* = 0.13 mm). *N*_*B*_ (=2) is the number of beams in each row and *N*_*rows*_ (=30) is the number of rows along the loading direction. The patterned region had lengths of *N*_*rows*_*w* $\cong$ 60, 90, 120 mm and widths of 2*l*_*M*_ + 3*d* $\cong$ 46, 66 mm. Pristine regions at both ends of kirigami patterns are clamped during testing. Three samples are tested under uniaxial tension for each design and geometry (Fig. 2a) using an Instron 5944 mechanical testing machine with a 50 N load cell at a displacement rate of 1 mm/s. Mechanical parameters are analyzed and calculated using MATLAB.

### Stretchable electronics demonstration

PET kirigami films are spray coated with an eutectic indium-gallium (EGaIn) electrode and thin copper film leads are attached to the plain regions of the kirigami sheets for electrical measurements. Kirigami films are clamped into the grips of an Instron 5944 as in the mechanical measurements and resistance is measured with a four-point probe method by attaching leads to the copper tape at each end of the sample. Electrical data is collected with a Keithley 2460 (Keithley Instruments. Inc.) and is synchronized with the mechanical measurements with a digital trigger. Furthermore, the electronics shown in Fig. 4c are fabricated by soldering LEDs onto copper film at each end of the sample.

### Kirigami actuator

The kirigami actuator is composed of a single layer of patterned magneto-elastomer composite. It is a 820 *μ*m thick film of polydimethylsiloxane (PDMS) elastomer (Sylgard 184 with a 10:1 base-to-curing agent ratio; Dow Corning) with 70% w/w 1 *μ*m Fe microparticles (US Research Nanomaterials Inc.). The sample is prepared by hand-mixing the PDMS and microparticles with a rod for 10 min followed by vacuum mixing in a planetary mixer (FlackTek Inc.; DAC 400.2 VAC) for complete dispersion. The Fe-PDMS mixture is cast onto a clean glass plate to form an even layer using a thin-film applicator (ZUA 2000; Zehntner Testing Instruments). This is cured at 70 °C for 4 h. and then patterned with a CO_2_ laser cutter to generate kirigami films. The magnetic field is generated by an electromagnet (Bunting Magnetics Co.) connected to a DC power supply providing a step function current of 4.0 A for 1 s and then off for 1 s. For high cycle testing, the magnet is powered off periodically to prevent overheating. The slow motion of the actuator was recorded at 240 fps with iPhone 6 and the recorded video was analyzed with a video analysis tool (Tracker; Open Source Physics).

## Electronic supplementary material

Video S1- Hybrid Kirigami Actuator

Supplementary Information
