# Supplementary material for: Tunable Mechanical Metamaterials through Hybrid Kirigami Structures
Source: Sci Rep. 2018 Feb 21;8:3378. doi: 10.1038/s41598-018-21479-7 (PMC5821861; doi:10.1038/s41598-018-21479-7)
Supplement: Supplementary file 2 — Supplementary Information [file 41598_2018_21479_MOESM2_ESM.pdf]

Supporting Information

Title: Tunable Mechanical Metamaterials through Hybrid Kirigami Structures

*Doh-Gyu Hwang and Michael D. Bartlett**

Department of Materials Science and Engineering, Soft Materials and Structures Lab, Iowa State University of Science and Technology, 528 Bissell Rd, Ames, IA 50011, USA

E-mail: mbartlet@iastate.edu

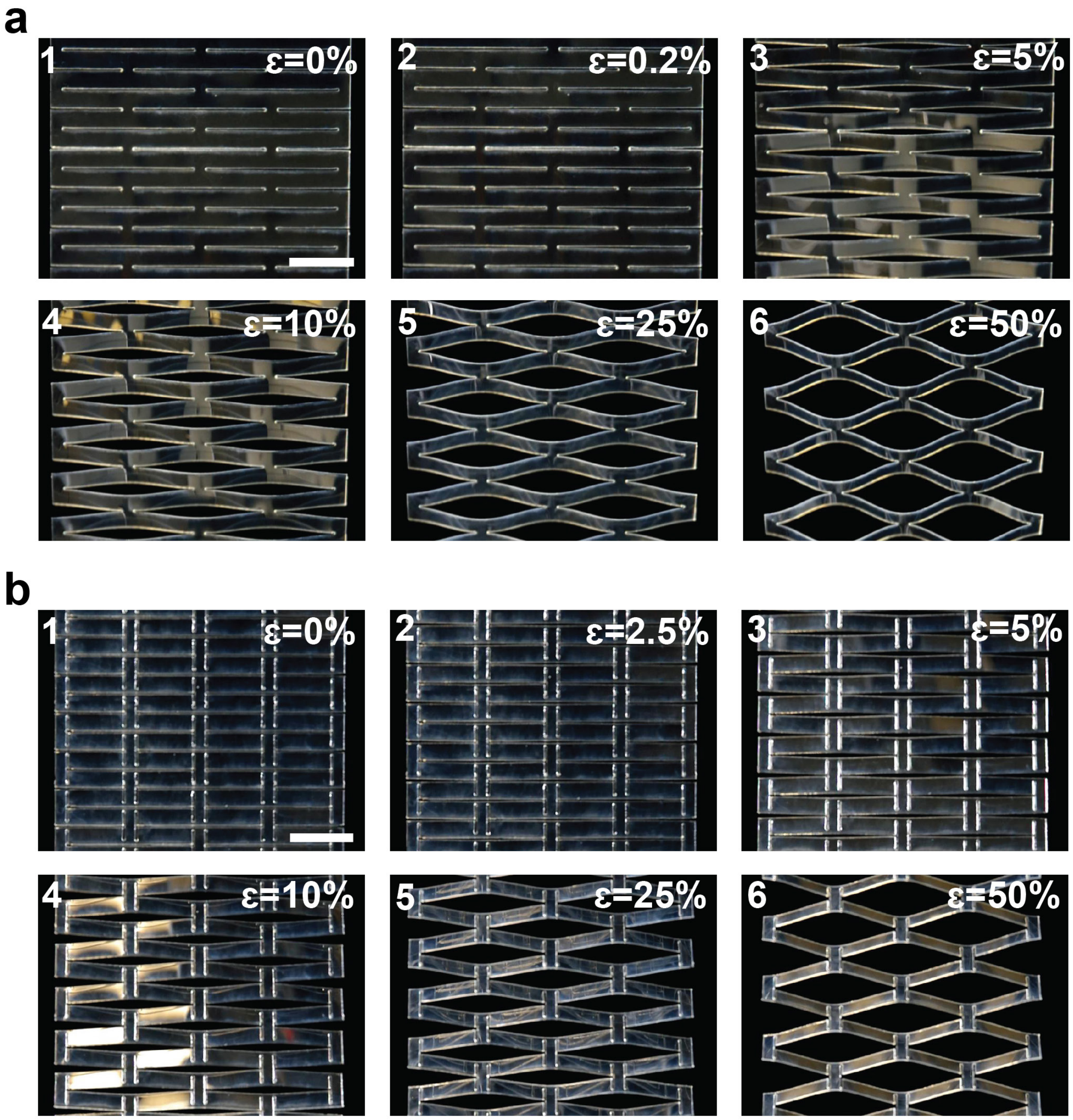

Figure S1: a) Image sequences of a krigiami structure without minor cuts (design *i*) and b) with minor cuts (design *v*) under uniaxial loading at the rate of 1mm/s. Scale bar : 10 mm. Geometric parameters in the structures are equivalent to those in Figure 1. The numbers in the top left corners of the image correspond to, 1 : Before tensile tests, 2: In-plane deformation, and 3-6 : Out-of-plane deformation.

Mechanical response of kirigami sheets

A sheet patterned with linear cuts can be regarded as an array of beams. Here we investigate the mechanical response of kirigami sheets with hybrid cut structures and provide analytical expressions for stiffness with different effective boundary conditions and non-prismatic beams/structures. We also derive analytical expressions for the ultimate strain behavior and provide data for the ultimate force when the kirigami sheets break under uni-axial loading.

Kirigami sheets with effective boundary conditions

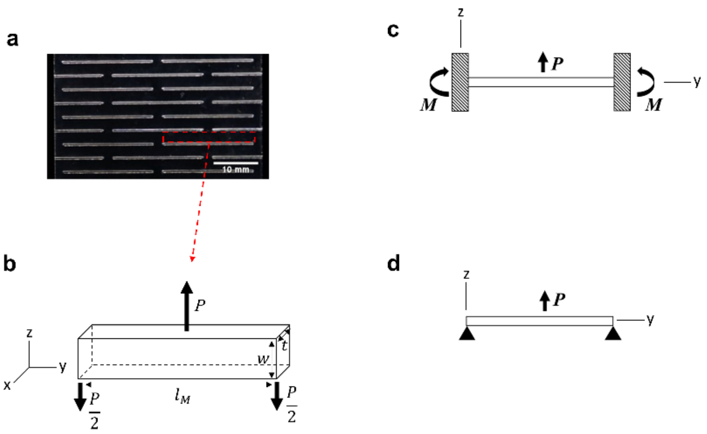

Figure S2: a) Kirigami film and b) schematic showing loading conditions. c) Fixed-fixed end conditions and d) pinned-pinned end conditions in a loaded beam.

Fixed-fixed end condition (FFE)

$$EI\frac{d^2\delta}{dx^2} = M(x) \tag{S1}$$

Where the bending moment is:

$$M(x) = \frac{P}{8}(4x - l_M) \quad \left(0 \leq x < \frac{l_M}{2}\right) \tag{S2}$$

With boundary conditions:

$$\frac{d\delta}{dx} = 0 \quad at\ x = \frac{l_M}{2} \quad and\ \delta = 0 \quad at\ x = 0 \tag{S3}$$

Where upon integration of equation S1 with boundary conditions in S3 we have the deflection at the midpoint, δ_C:

$$\delta_C = \frac{Pl_M^3}{192EI} \tag{S4}$$

With $I = w^3t/12$, we obtain the stiffness (k):

$$k = 16\frac{Ew^3t}{l_M^3} \tag{S5}$$

Where the numerical coefficient (α) is equal to $\alpha = 16$, which is dependent on boundary conditions and beam shape.

Pinned-pinned end condition (PPE)

$$EI\frac{d^2\delta}{dx^2} = M(x) \tag{S6}$$

Where the bending moment is:

$$M(x) = \frac{P}{2}x \quad \left(0 \leq x < \frac{l_M}{2}\right) \tag{S7}$$

With boundary conditions:

$$\frac{d\delta}{dx} = 0 \quad at\ x = \frac{l_M}{2} \quad and\ \delta = 0 \quad at\ x = 0 \tag{S8}$$

Where upon integration of equation S1 with boundary conditions in S2 we have the deflection at the midpoint, δ_C:

$$\delta_C = \frac{Pl_M^3}{48EI} \tag{S9}$$

With $I = w^3t/12$, we obtain the stiffness (k):

$$k = 4\frac{Ew^3t}{l_M^3} \tag{S10}$$

Where the numerical coefficient (α) is equal to $\alpha = 4$, which is dependent on boundary conditions and beam shape.

Kirigami sheets consisting of non-prismatic beams

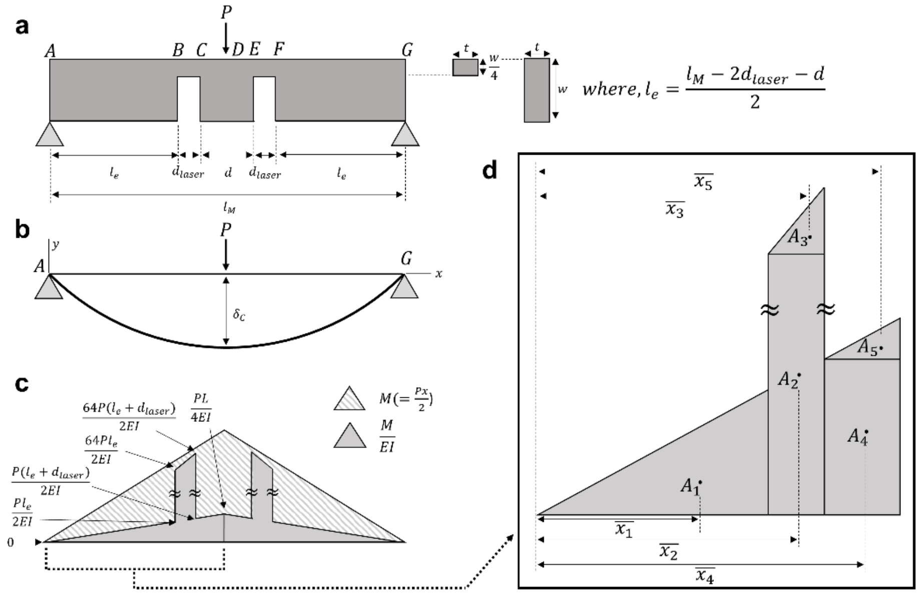

Figure S3: a) Schematic of non-prismatic beam with defined geometry and b) beam under load. c) M/EI diagram and d) detailed view of sections.

For a non-prismatic beam, where the flexural rigidity *EI* is not constant along the length, the stiffness is calculated using the moment-area method. Here we adopted a specific system with the following dimensions: $l_M = 20mm, l_e = 8.85mm,\ d_{laser} = 0.3mm,\ d = 0.85mm,\ I_{AB,CD} = 64I_{BC}$. The area of the M/EI diagram, A_i, for the different segments of the beam are:

$$A_1 = \frac{1}{2}(0.4425l_M)\left(\frac{P(0.4425l_M)}{2EI}\right) \quad A_2 = (0.015l_M)\left(\frac{64P(0.4425l_M)}{2EI}\right)$$

$$A_3 = \frac{1}{2}(0.015l_M)\left(\frac{64P(0.015l_M)}{2EI}\right) \quad A_4 = (0.0425l_M)\left(\frac{P(0.4575l_M)}{2EI}\right) \tag{S11}$$

$$A_5 = \frac{1}{2}(0.0425l_M)\left(\frac{P(0.0425l_M)}{2EI}\right)$$

The displacement is then calculated as:

$$\delta_C = A_1\bar{x}_1 + A_2\bar{x}_2 + A_3\bar{x}_3 + A_4\bar{x}_4 + A_5\bar{x}_5 \tag{S12}$$

where $\bar{x}_1 \sim \bar{x}_5$ are the distances between point A and the centroids of individual areas. These distances are:

$$\bar{x}_1 = \frac{2}{3}(0.4425l_M); \quad \bar{x}_2 = 0.4425l_M + \frac{1}{2}(0.015l_M); \tag{S13}$$

$$\bar{x}_3 = 0.4425 l_M + \frac{2}{3}(0.015 l_M); \quad \bar{x}_4 = 0.4575 l_M + \frac{1}{2}(0.0425 l_M);$$

$$\bar{x}_5 = 0.4575 l_M + \frac{2}{3}(0.0425 l_M)$$

The deflection at the midpoint, δ_C is then:

$$\delta_C = 0.116523 \frac{P l_M^3}{EI} \tag{S14}$$

With $I = w^3 t/12$, we obtain the stiffness (k):

$$k \cong 0.7 \frac{E w^3 t}{l_M^3} \tag{S15}$$

Where the numerical coefficient (α) is equal to $\alpha \approx 0.7$. For the case of a $l_M = 30$ mm beam, $\alpha \approx 0.9$. In the manuscript, both $l_M = 20$ mm and $l_M = 30$ mm beams are displayed on the same plot, for clarity of presentation we choose $\alpha \approx 0.8$ when displaying theoretical predictions.

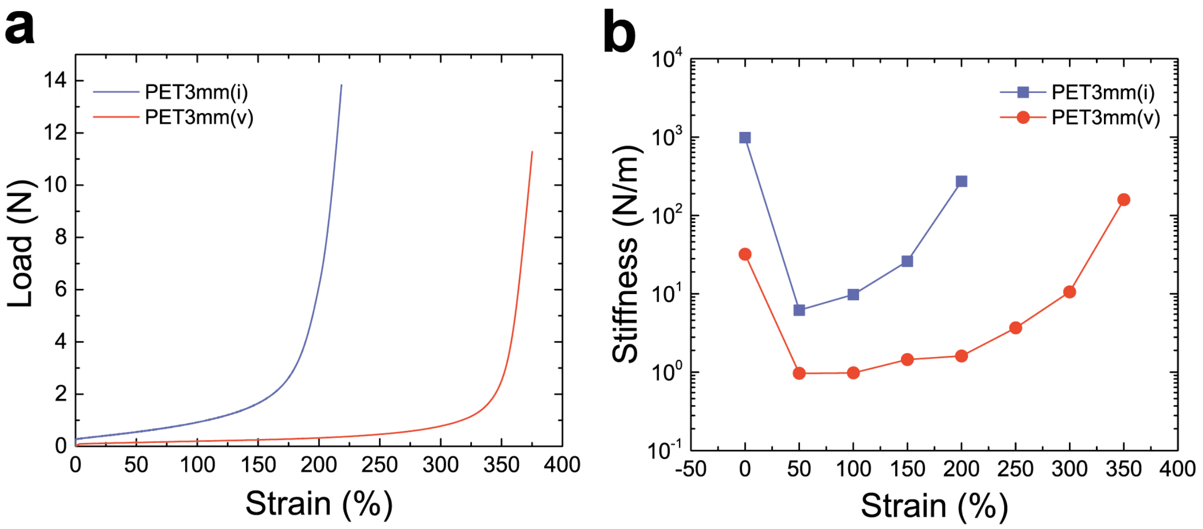

Figure S4: a) Load (P) versus strain (ε) for kirigami materials with design i of w = 3mm (blue curve) and with design v of w = 3mm and $l_m/2w = 0.75$ (red curve). See Figure 1b to see a close-up view of the initial regime of both patterned structures of the $P - \varepsilon$ plot. The slope indicates the effective in-plane stiffness. b) Log-linear plot of Stiffness versus strain for equivalent designs and geometric parameters. Effective in-plane stiffness ($\varepsilon = 0\%$) and effective out-of-plane stiffness ($\varepsilon > 0\%$) are calculated by linearly fitting experimental data points with a 5 % strain window every 50%.

Derivation of ultimate strain for kirigami sheets

To predict ultimate strain where kirigami sheets break, we derive an empirical formula based on work by Isobe *et al* and Shyu *et al*.[28,29] Their approach assumes that all the beams deform in the same manner. However, when the number of rows N_{rows} along the axial direction is finite, this can overestimate ε_{ULT} due to edge effects. At the edge of the sample there is a region of reduced displacement, R_1, compared to regions of uniform displacement, R_2, away from the edges (See Figure S3). Experimentally, we find the reduction of displacement in the initial and final regimes to be $R_1/R_2 \approx 0.7$, as summarized in Table S1. In addition, each beam cannot perfectly align along the loading direction due to the connectivity between beams. This results in a finite angle between the beam and the loading direction, as seen in Figure S3, which upon performing image analysis on samples stretched to ε_{ULT}varies between $\theta \approx 0° \sim 22°$ in our system for $l_M \geq 5w$ (see Table S1 for details).

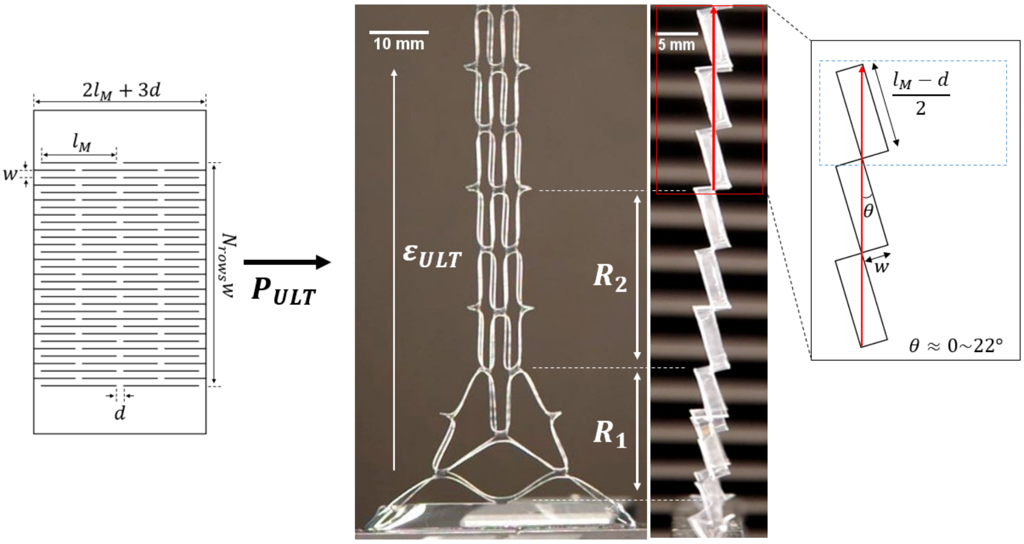

Figure S5: Stretching of kirigami film without minor cuts to examine ultimate stretching behavior.

w/l_M	2/30	2/20	3/20	4/20
	29.35/43.74	22.12/30.30	22.46/30.58	23.40/32.78
R_1/R_2	30.14/44.98	21.71/30.40	22.22/31.04	23.77/32.10
	30.68/45.01	21.82/30.38	21.78/30.55	22.90/32.08
θ	8°	10°	18°	22°
Average	0.674	0.721	0.721	0.723

Table S1: Edge effects and beam angle in direction of loading at ε_{ULT}.

When taking into account edge effects and the finite angle upon maximum extension we find that the ultimate length of the sample, L_{ULT}, can be calculate as:

$$L_{ULT} = N_{rows}\sqrt{w^2 + \left(\frac{l_M - d}{2}\right)^2} - 2 \times N_{rows,edg} \times \left(1 - \left(\frac{R_1}{R_2}\right)\right)\sqrt{w^2 + \left(\frac{l_M - d}{2}\right)^2} \quad \text{(S16)}$$

Or

$$L_{ULT} = N_{rows}\frac{l_M - d}{2\cos\theta} - 2 \times N_{rows,edg} \times \left(1 - \left(\frac{R_1}{R_2}\right)\right)\frac{l_M - d}{2\cos\theta} \quad \text{(S17)}$$

Where $N_{rows,edg}$ is the number of rows under the influence of edge effects. According to images of samples throughout experiments, the first 3 and last 3 rows show significant edge effect ($N_{rows,edg} = 3$). The numerical value 2 before $N_{rows,red}$ takes into account both end regimes along the loading direction. When $1 - (R_1/R_2) \approx 0.3$, Equation S17 and S18 can be simplified with the assumption that $N_{rows,edg} \times (1 - (R_1/R_2)) \approx 1$:

$$L_{ULT} = (N_{rows} - 2)\frac{l_M - d}{2\cos\theta} \quad \text{(S18)}$$

With $L_0 = N_{rows}w$, the ultimate strain can be defined as:

$$\varepsilon_{ULT} = \frac{\Delta L}{L_0} = \frac{L_{ULT} - L_o}{L_o} = \frac{(N_{rows} - 2)\frac{l_M - d}{2\cos\theta}}{N_{rows}w} - 1 \quad \text{(S19)}$$

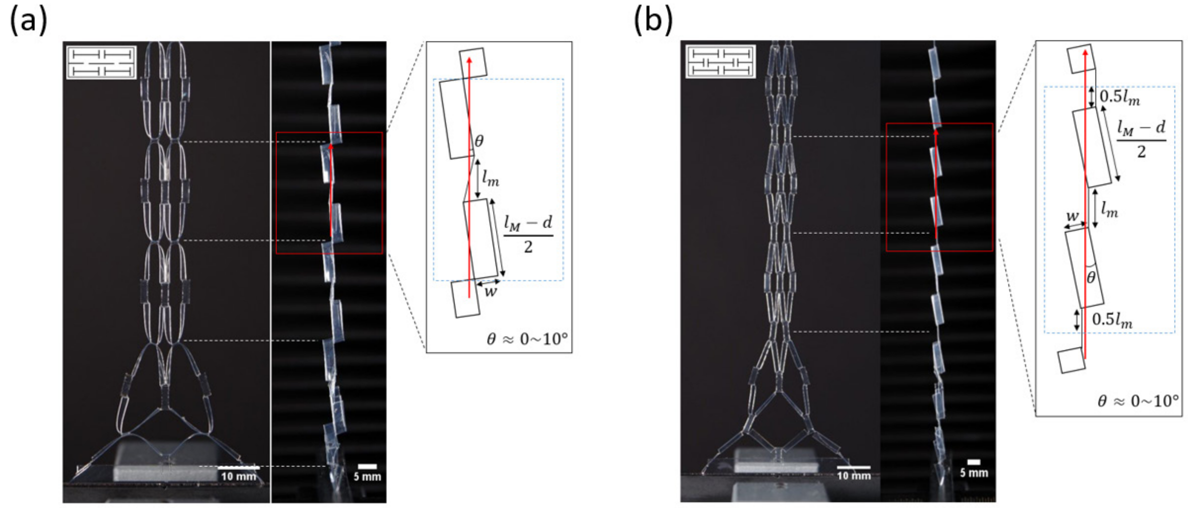

Figure S6: Stretching of kirigami film with minor cuts in a) alternating rows and b) every row to examine ultimate stretching behavior.

In our system, we introduce minor cuts into a kirigami structure containing major cuts. This modification leads to a change in ultimate strain due to the opening up of minor cuts by a factor of γl_m, where $\gamma = 0, 1, 2$ for no minor cuts, minor cuts on alternating rows, and minor cuts in every row respectively and l_m is the length of the minor cut. Thus, the ultimate length of the sample, L_{ULT}, can be calculated as:

$$L_{ULT} = (N_{rows} - 2)\left(\frac{l_M - d + \gamma l_m}{2\cos\theta}\right) \tag{S20}$$

With $L_0 = N_{rows}w$, the ultimate strain is then:

$$\varepsilon_{ULT} = \frac{\Delta L}{L_o} = \frac{(N_{rows} - 2)\left(\frac{l_M - d + \gamma l_m}{2\cos\theta}\right)}{N_{rows}w} - 1 \tag{S21}$$

If the kirigami structure is composed of slender beams with relatively short spacing such that $l_M \gg w, d$ so $\cos\theta \to 1$, and the number of rows is large so that $N_{rows} - 2 \approx N_{rows}$, equation S21 can be simplified to:

$$\varepsilon_{ULT} \approx \frac{l_M + \gamma l_m}{2w} - 1 \tag{S22}$$

We have adopted this simplified equation when calculating $\frac{\varepsilon_{ULT}}{\bar{K}}$. This can also be examined as the ultimate stretch ratio, $\lambda_{ULT} = L_{ULT}/L_o$, and can be calculated as $\lambda_{ULT} \approx \frac{l_M+\gamma l_m}{2w}$.

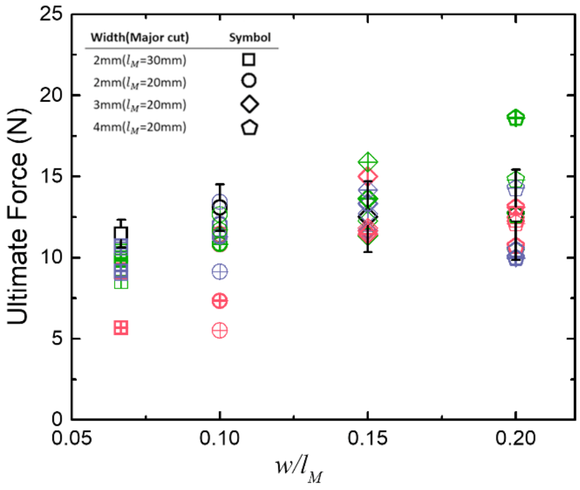

Figure S7: Ultimate force of kirigami materials. See Figure 2a for key to symbol color and fill pattern.
